# Automatic Registration of Homogeneous and Cross-Source TomoSAR Point Clouds in Urban Areas

**DOI:** 10.3390/s23020852

**Published:** 2023-01-11

**Authors:** Lei Pang, Dayuan Liu, Conghua Li, Fengli Zhang

**Affiliations:** 1 School of Geomatics and Urban Spatial Informatics, Beijing University of Civil Engineering and Architecture, Beijing 102616, China; 2 Aerospace Information Research Institute, Chinese Academy of Sciences, Beijing 100094, China

**Keywords:** homologous TomoSAR point cloud, cross-source TomoSAR point cloud, the normal vector of the opposite facade, the facade projection

## Abstract

Building reconstruction using high-resolution satellite-based synthetic SAR tomography (TomoSAR) is of great importance in urban planning and city modeling applications. However, since the imaging mode of SAR is side-by-side, the TomoSAR point cloud of a single orbit cannot achieve a complete observation of buildings. It is difficult for existing methods to extract the same features, as well as to use the overlap rate to achieve the alignment of the homologous TomoSAR point cloud and the cross-source TomoSAR point cloud. Therefore, this paper proposes a robust alignment method for TomoSAR point clouds in urban areas. First, noise points and outlier points are filtered by statistical filtering, and density of projection point (DoPP)-based projection is used to extract TomoSAR building point clouds and obtain the facade points for subsequent calculations based on density clustering. Subsequently, coarse alignment of source and target point clouds was performed using principal component analysis (PCA). Lastly, the rotation and translation coefficients were calculated using the angle of the normal vector of the opposite facade of the building and the distance of the outer end of the facade projection. The experimental results verify the feasibility and robustness of the proposed method. For the homologous TomoSAR point cloud, the experimental results show that the average rotation error of the proposed method was less than 0.1°, and the average translation error was less than 0.25 m. The alignment accuracy of the cross-source TomoSAR point cloud was evaluated for the defined angle and distance, whose values were less than 0.2° and 0.25 m.

## 1. Introduction

As an extension of the interferometric synthetic aperture radar (InSAR) technology, the synthetic aperture principle is extended to the elevation direction, solving the overlay mask problem caused by the SAR imaging geometry, and realizing the three-dimensional imaging of the distance direction, azimuth direction, and elevation direction [1,2]. In recent years, with the improvement of airborne SAR and satellite-based SAR systems and the advancement of technology, the resolution, signal-to-noise ratio, and other indices have been improved, and high-precision 3D building point clouds in the observation area can now be generated using airborne or satellite-based SAR tomography technology, while even higher-dimensional information such as building deformation can be obtained using differential tomography technology [3,4,5].

The three-dimensional visualization of urban buildings plays an extremely important role in the process of urban digital construction. Since the synthetic aperture radar is side-imaging, the TomoSAR point cloud generated by a single track only shows the structure of one side of the building, and the TomoSAR point cloud generated by SAR images of at least two tracks is needed to show the complete structure of the building in the target area. The team of Zhu Xiaoxiang [6] fused the TomoSAR point cloud of the ascending and descending orbit of the Berlin urban area for the first time, and the fused TomoSAR point cloud could realize the construction of urban dynamic models and 3D visualization. However, in the environment of urban expansion, the low coherence and noise of buildings in the observation area lead to a reduction in the total amount of SAR images and the quality of TomoSAR point clouds, thus limiting the application of spaceborne SAR 3D imaging. Due to the different number of SAR images in different orbits and the error of geocoding, there are some rotation and translation errors in multi-view TomoSAR point clouds. The 3D visualization of a complete building structure based on the TomoSAR point cloud can not only rely on the TomoSAR point cloud generated by the SAR image of rising and falling tracks but also be realized by combining TomoSAR and LiDAR point clouds. The backpack mobile 3D laser scanner uses the laser SLAM principle, and the operation is very simple [7,8]. It restores the spatial 3D data through the algorithm as a function of its attitude data and laser point cloud. The detection distance of the backpack mobile 3D laser scanner is 50–120 m. Scanning a high-rise building of more than 100 m can easily cause the loss of the facade and top point cloud of the high-rise building; in contrast, SAR is prone to missing point clouds at the bottom of buildings in complex environments, but it can detect the upper floors and top areas of buildings. Compared with TomoSAR point clouds, mobile laser scanning (MLS) point clouds have higher density and a very low overlap rate. However, how to extract the same features to fuse ascending and descending TomoSAR point clouds with single-track TomoSAR and MLS point clouds is the main research problem addressed in this paper.

Point cloud automatic registration mainly adopts the registration strategy from coarse to fine. Firstly, the rough point cloud registration algorithm is used to roughly estimate the altitude conversion parameters between the two-point cloud data, i.e., the initial rotation and translation parameters. Then, the initial conversion parameters are used as the input parameters of the point cloud precision registration algorithm to further accurately register the two-point clouds, and a higher precision point cloud registration result is obtained [9,10,11]. The main features used for point cloud automatic coarse configuration are point [12,13], line [10,14], and face [15,16]. Point features such as SIFT [17,18], Harris [19,20], and FPFH [21,22] are extracted for automatic registration of airborne laser scanned (ALS) and terrestrial laser scanned(TLS) building point cloud data [10,23]. Extracting line and surface features for automatic registration leads to higher robustness than point features, and it can effectively reduce the interference of point cloud noise [11]. The 2-D contours of buildings were extracted to automatically register ground and airborne point clouds in [24]. A parameterization based on complex numbers was used to determine the corresponding relationship between planes, which was effectively applied to the ground laser scanning data with a certain degree of overlap.

At present, the commonly used methods of point cloud precise registration are the iterative nearest neighbor algorithm (ICP), random sampling consistency (RANSAC), normal distribution transformation algorithm (NDT), etc. Among them, the ICP algorithm [25] iteratively corrects the rigid body transformation (translation and rotation) of two original point clouds to minimize the distance between all point sets. The RANSAC algorithm [26] achieves this goal by iteratively selecting a set of random subsets of point cloud data with a certain probability to get a reasonable alignment result, and the number of iterations must be increased to improve the probability. The NDT algorithm [27] uses the statistical information of the point cloud data, whereby the probability density of the transformed points is maximized if the transformed parameters are the best alignment result of the two point clouds.

The above methods are commonly used in the coalignment of LiDAR point clouds and point clouds derived from optical images, but they are not applicable to point clouds derived from SAR images because the overlap rate of TomoSAR point clouds generated from cross-directional orbits is extremely low, and the density and accuracy of point clouds are low compared with LiDAR point clouds; hence, the automatic alignment of TomoSAR point clouds faces greater difficulties. Gernhardt et al. [28] used the fused PSI for detailed monitoring of individual buildings. In [6], the automatic alignment of the TomoSAR point clouds of ascending and descending orbits was achieved by extracting the L-shaped endpoints of the TomoSAR building facade point clouds, and the L-shaped endpoints of the TomoSAR point clouds of the two orbits could not be accurately corresponded when there were fewer point clouds of one of the building L-shaped facades. Hence, this method was limited to buildings with L-shaped facades. A robust alignment method for urban area array InSAR point clouds was proposed in [29], using the concave and convex facades of buildings for rotation correction and fine displacement. The TomoSAR and MLS point clouds in urban areas also have rich facade information. In this paper, the geometric features of the TomoSAR and MLS facade point clouds were used to derive the optimal alignment parameters, i.e., rotation matrix and translation matrix, to achieve automatic alignment between homologous and cross-source TomoSAR point clouds. Thus, the main contributions of the work in this paper are as follows:

- A method is proposed for aligning TomoSAR point clouds for both ascending and descending orbits, and TomoSAR point clouds with MLS point clouds;
- Rotation correction is performed using the normal vector angle of the opposing facades of the building;
- Fine translation correction of the spatial position of the opposing facades of the building is achieved using previous information.

## 2. Materials and Methods

**Figure 1:**
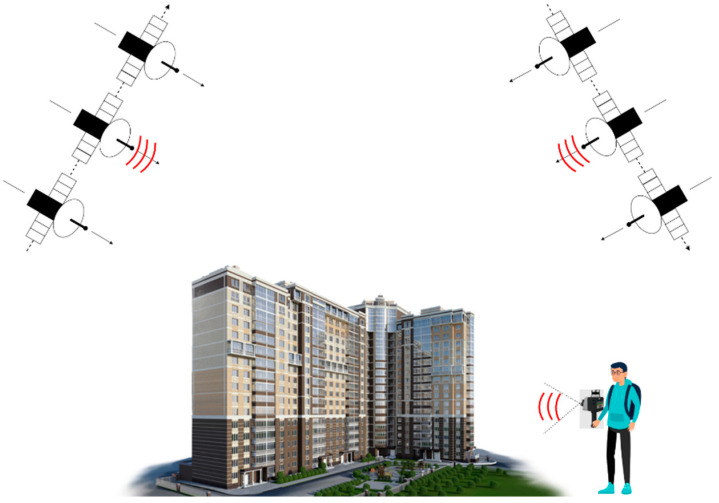
Schematic map of multi-view urban building point cloud acquisition (tomographic synthetic aperture radar system and backpack mobile 3D laser scanner).

Three-dimensional point clouds were acquired from two different views of the urban area, as shown in Figure 1. For the ascending and descending TomoSAR point clouds, the homologous TomoSAR point clouds had offset and rotation errors perpendicular to the line of sight due to the satellite platform position and geocoding errors, and the overlap rate of the two point clouds was extremely low. For TomoSAR point clouds and MLS point clouds, the point cloud densities were different, and the two point clouds obtained from different viewpoints belonged to the opposite facades of buildings; therefore, the overlap information could not be used for alignment fusion. Accordingly, we propose an alignment method using the point cloud characteristics of building facades.

**Figure 2:**
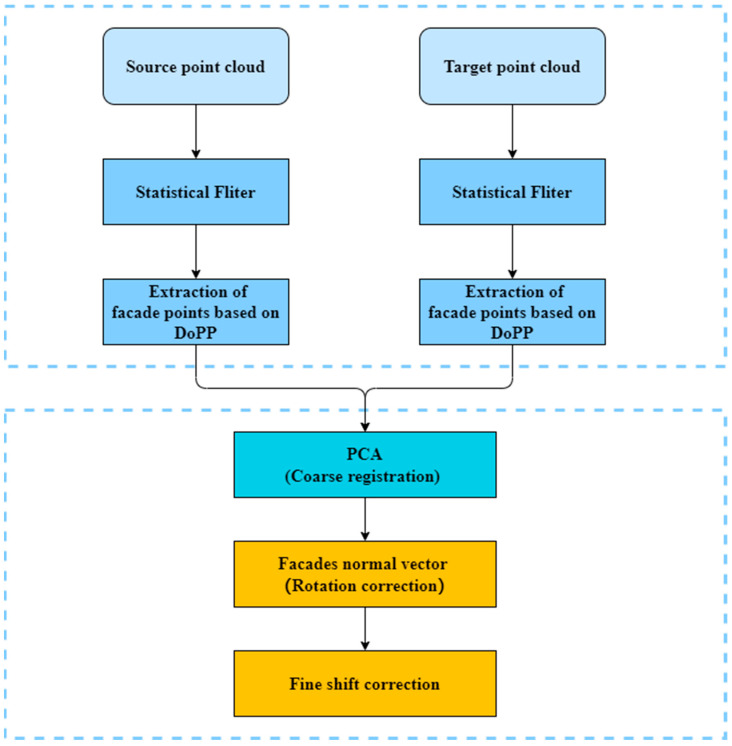
Flowchart of the proposed method. The rectangular box in the upper half represents the preprocessing of the data: filtering and extraction of building elevation points. The rectangular box in the lower half represents the step of data alignment: coarse alignment followed by rotation and fine translation correction.

Our method was implemented in C++ based on the existing functions of PCL. All the experiments are conducted on a computer with an Intel i7-11700 and 32-GB RAM. The flow chart of the method is shown in Figure 2. The source and target point clouds were composed of the TomoSAR point clouds of the ascending and descending orbits, or the source and target point clouds were composed of the single-orbit TomoSAR point cloud and the MLS point cloud, and the two point clouds provided the front and reverse sides of the building. Firstly, statistical filtering was used for filtering, and most of the noise and outlier points were eliminated. The filtered point cloud still had some of the denser outlier block point clouds. According to the DoPP algorithm, to extract the building facade points, the extracted building facade points were clustered by density to obtain the building facade blocks, thus further eliminating the outlier points. To ensure that the source and target point clouds had good initial positions, the PCA-based initial coarse alignment method was used, which mainly used the principal axis direction of the point cloud data to align the two sets of point clouds with good initial positions after alignment. Using RANSAC to fit the building facade points to get the plane of the building facade, the normal vector of the plane, angle of the normal vector according to the topological relationship with the building facade, and rotation coefficient were sequentially calculated. After projecting the fitted plane point cloud onto the *xy*-axis, the least squares method was used to fit a two-dimensional straight line, and the translation coefficient was calculated according to the spatial position of the building facade.

The method of data pre-processing described above involves several artificially given parameters, including statistical filtering, building point extraction, and RANSAC-based façade extraction. We set these parameters in combination with building spacing, number of building storys and point cloud density:

- Statistical filtering: the number of close points analyzed for each point is set to 50 and the multiple of the standard deviation is set to 1. This means that a point is marked as an outlier if it exceeds the mean distance by more than one standard deviation;
- Building elevation point extraction: Set the grid size of the DoPP projection to 0.5 m × 0.5 m, and set the number of points of a single grid to 15 for TomoSAR facade point cloud estimation;
- Facade extraction based on RANSAC: As the remaining thickness of the building facade points is about 1~2 m, the facade plane fitting tolerance is set to 0.5 m, resulting in an average facade thickness of 1.5 m.

### 2.1. TomoSAR System Model

**Figure 3:**
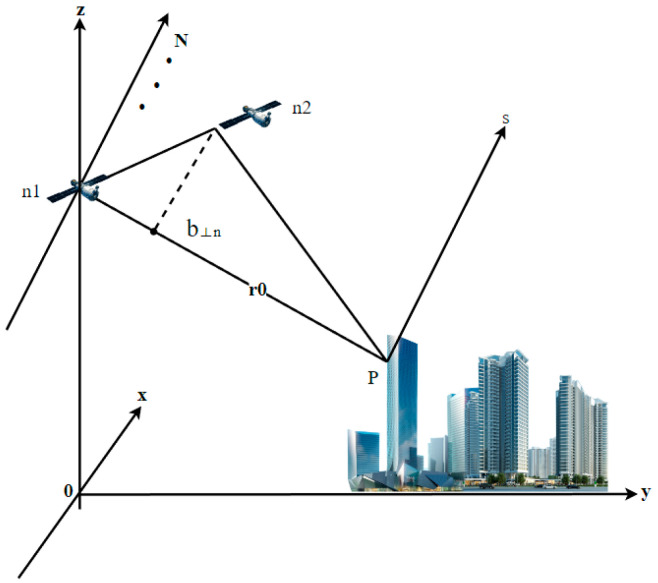
The imaging mode of TomoSAR.

TomoSAR, which originated from medical CT imaging technology, extends the two-dimensional imaging principle of SAR to three dimensions. TomoSAR uses multiple aligned two-dimensional SAR images obtained from observations of the same target feature to invert its scattering values at different heights in the oblique distance direction, thus restoring the real three-dimensional scene [30,31]. The geometric model of the TomoSAR imaging principle is shown in Figure 3. One of the M + 1 view aerial pass SAR single-view complex images of the same target area was selected as the main image, and the complex value gm of each resolution unit in the m-th aerial pass image except the main image could be regarded as the superposition of N scattered target signals in the same orientation at the same oblique distance in the laminar direction s. This can be expressed as follows:

(1) $$g_{n}=\int_{\Delta s}^{\;}\gamma \left(s\right)exp\left(-j2\pi \xi_{n}s\right)ds,n=1,2,3,\ldots ,N,$$

where γ(*s*) is the backward reflectivity function along the elevation direction of the imaging area, and the spatial sampling interval $\xi_{n}$ can be calculated as $\xi_{n}=-2b_{⊥n}/\left(\lambda r\right)$, $b_{⊥n}$ is the vertical baseline distance, λ is the incident wavelength, Δs is the range of elevation angles depending on the width of the antenna diffraction pattern, and r is the central slope distance. After discretization, Equation (1) can be simply approximated as follows:

(2) g = Rγ+ε

where g is the measurement vector of length N, R is the dictionary matrix with size N × L, L is the number of grid cells divided on the s-axis,$\;A_{ik}=exp\left(-j2\pi \xi_{k}s_{k}\right)$ is the element of the i-th row and k-th column of the matrix, and ε is the noise vector.

According to linear algebra theory, Equation (2) becomes an underdetermined equation with a nonunique solution space when the number of samples in the elevation direction is much larger than the actual number of coherent trajectories. A common solution is to use compressed sensing methods [32]. The objective function with a sparse constraint term is as follows:

(3) $$\hat{\gamma }=arg\;\underset{x}{\min}\left\{∥R\gamma -g{∥}_{2}^{2}+\lambda {∥\gamma ∥}_{1}\right\}$$

where λ denotes the sparsity factor. A larger value indicates a sparser solution. ${∥\gamma ∥}_{1}$ is the sparsity constraint term that limits the solution space.

### 2.2. Filtering and Facade Point Extraction

The TomoSAR point cloud of urban scenes had more outliers; in order to extract the building facade points effectively, statistical filtering was first used to remove obvious outliers. The outliers were sparsely distributed, and the distances of all points in the point cloud formed a Gaussian distribution. The average distance of each point to its nearest k points was calculated, and the mean and variance were designed to eliminate the outliers smaller than the set value.

For both TomoSAR and MLS point clouds, the building facade points could be extracted on the basis of the density of the projection point, whereby the point cloud is divided using a horizontal grid, and the number of projection points falling on each grid cell is counted. For the characteristics of TomoSAR point clouds, the DoPP values were much larger in the building facade than in other areas. The DoPP values of noise points caused by multiple scattering and noise points on the ground were uniform and small; for the point clouds on top of buildings and ground features, the DoPP values were locally larger. Using the above characteristics, a reasonable threshold value could be selected to classify the TomoSAR point clouds, with DoPP greater than T1 for the building facade point clouds, DoPP less than T2 for the noise points, and the remaining DoPP for the point clouds of the top of buildings and ground features [33].

**Figure 4:**
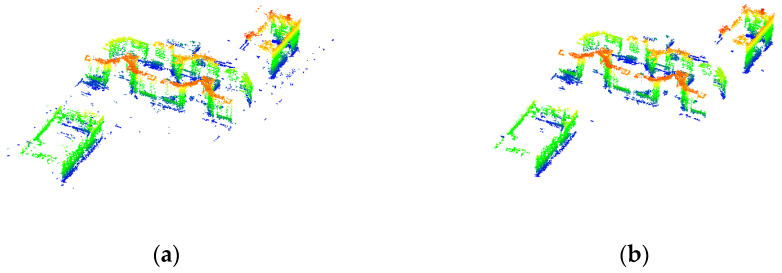

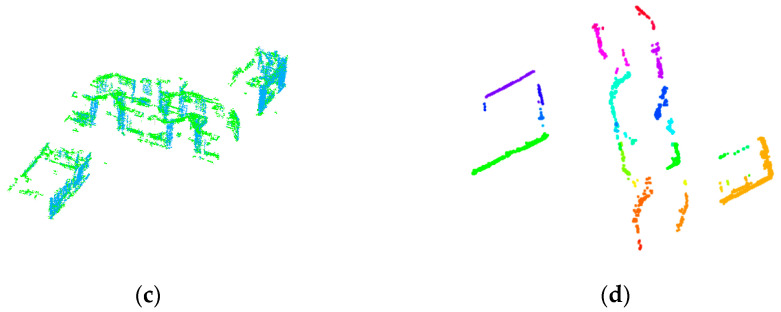
Results of filtering and façade point extraction: (**a**) original point cloud; (**b**) point cloud based on statistical filtering; (**c**) building points extracted according to DoPP projection, with blue indicating building façade points and green indicating building planes or other structural points; (**d**) results of density clustering.

Figure 4b shows the statistically filtered point cloud with most of the outlier points removed. Figure 4c shows the results of extracting the elevation points according to the DoPP projection point density, where the blue point cloud is the building elevation point and the green point cloud is composed of the small-scale building points and the top point cloud. In Figure 4d, the observations in the *n* × *p* data matrix X are divided into clusters according to the DBSCAN algorithm, and the extracted building facade points are partitioned into point cloud blocks by clusters.

### 2.3. Coarse Alignment

Point cloud alignment was divided into two steps: coarse alignment and fine alignment. Coarse alignment referred to when the transformation between two point clouds was unknown, aimed at providing a better initial value of transformation for the fine alignment; the fine alignment criterion was given an initial transformation and further optimized to obtain a more accurate transformation.

For rigidly transformed point cloud alignment, the transformation factor T can be expressed as follows [34]:

(4) $$T=\left|\begin{array}{c} \begin{array}{cc} R_{11} & R_{12}\\ R_{21} & R_{22} \end{array}\;\begin{array}{cc} R_{13} & x'\\ R_{23} & y' \end{array}\\ \begin{array}{cc} R_{31} & R_{32}\\ 0 & 0 \end{array}\;\begin{array}{cc} R_{33} & z'\\ 0 & 1 \end{array} \end{array}\right|,R^{*}=\left|\begin{array}{ccc} R_{11} & R_{12} & R_{13}\\ R_{21} & R_{22} & R_{23}\\ R_{31} & R_{32} & R_{33} \end{array}\right|,t^{*}=\left|\begin{array}{c} x'\\ y'\\ z' \end{array}\right|$$

where $R^{*}$ and $t^{*}$ are the rotation and translation coefficients.

**Figure 5:**
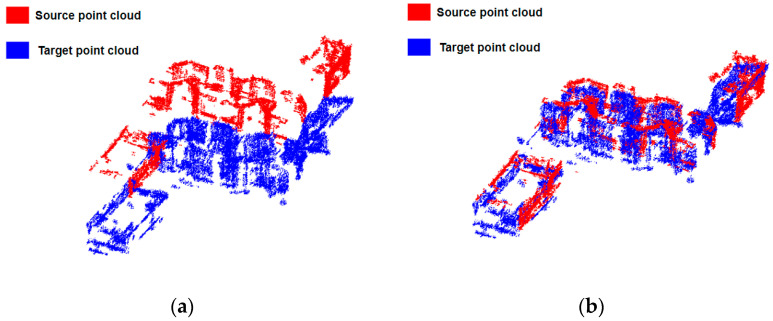
(**a**) The initial positions of the source and target point clouds. (**b**) The two point clouds after coarse alignment based on PCA.

The PCA-based initial alignment method mainly uses the principal axis direction of the extracted façade point cloud data for alignment [35]. Firstly, the covariance matrix of the two sets of point clouds is calculated, and the main feature components, i.e., the principal axis directions of the point cloud data, are calculated according to the covariance matrix. Then, the rotation matrix is derived from the principal axis direction, and the translation vector is directly derived by calculating the translational shift of the center coordinates of the two sets of point clouds. As shown in Figure 5a, the source and target point clouds were not parallel and had rotation and translation errors. Figure 5b shows the results after coarse alignment based on PCA, where the two point clouds had good initial spatial positions after coarse alignment but still have some rotation and translation errors. Therefore, a rotation and translation correction using the characteristics of the point cloud of the building façade is proposed below in order to recover the correct spatial position between the building facades.

### 2.4. Rotation Correction

**Figure 6:**
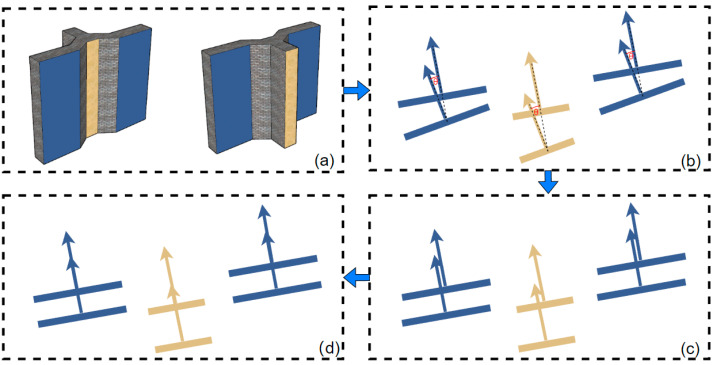
Schematic diagram of the spatially positioned fusion point cloud process using pairs of building elevations. (**a**) Ideal paired target and source facades with the same color facade spatial positions parallel. (**b**) The arrows indicate the corresponding normal vectors of the facades, and the normal vectors are not parallel with obvious pinch angles. (**c**) The normal vectors of the opposing elevations are parallel after rotation correction of the facade positions. (**d**) Exact translation correction of the facade position.

The easiest way to fit the plane is least squares fitting, but the accuracy of least squares fitting is easily affected by noise, while the random sample consensus (RANSAC) algorithm is a method to calculate mathematical model parameters from a series of data containing outliers. By fitting the plane with RANSAC, the effect of noise can be excluded, and the fitting accuracy can be greatly improved [36]. As shown in Figure 6a, the facades of the same color were the opposite facades of the same building, and the point clouds of the opposite facades were fitted using RANSAC after coarse alignment.

When the height or width of the fitted elevation is close to that of its opposite elevation, the normal vector of the plane should be calculated to perform the rotation correction. The eigenvector corresponding to the minimum eigenvalue of the covariance matrix calculated by PCA is the normal vector of the plane. Since the eigenvectors calculated by PCA are dualistic, the normal vectors of the opposing facades of the building are oriented such that the normal vectors of the two planes are oriented in the same direction. The angle between the normal vectors of the opposing faces of the building should be 0°. As shown in Figure 6b, the normal vectors of the opposing facades were not parallel and had a certain angle; thus, the angle between the vectors and the rotation axis could be found according to the two normal vectors. The formula for the rotation matrix was derived as described below.

It is known that the vector before rotation is $\vec{a}\left(a_{1},a_{2},a_{3}\right)$ and the vector after rotation is $\vec{b}\left(b_{1},b_{2},b_{3}\right)$; hence, the vector inner product is

(5) $$\vec{a}\cdot \vec{b}=\left|\vec{a}\right|\left|\vec{b}\right|\cos\theta$$

The angle between the vectors $\vec{a}$ and $\vec{b}$ is

(6) $$\theta =arccos\left(\frac{\vec{a}\cdot \vec{b}}{\left|\vec{a}\right|\left|\vec{b}\right|}\right)$$

Following cross-multiplication,

(7) $$a\times b=\left(a_{2}b_{3}-a_{3}b_{2}\right)i+\left(a_{3}b_{1}-a_{1}b_{3}\right)j+\left(a_{1}b_{2}-a_{2}b_{1}\right)k$$

Then, the rotation axis $\vec{c}$ is

(8) $$\left(\begin{array}{c} c_{1}\\ \begin{array}{c} c_{2}\\ c_{3} \end{array} \end{array}\right)=\left(\begin{array}{c} a_{2}b_{3}-a_{3}b_{2}\\ \begin{array}{c} a_{3}b_{1}-a_{1}b_{3}\\ a_{1}b_{2}-a_{2}b_{1} \end{array} \end{array}\right)$$

The rotation matrix $R^{*}$ is obtained from the Rodrigues rotation formula [37,38]:

(9) $$R^{*\;}=E\cos\theta +\left(1-\cos\theta \right)\left(\begin{array}{c} a_{1}\\ \begin{array}{c} a_{2}\\ a_{3} \end{array} \end{array}\;\right)\left(a_{1},a_{2},a_{3}\right)+\sin\theta \left(\begin{array}{c} 0\\ \begin{array}{c} a_{3}\\ -a_{2} \end{array} \end{array}\begin{array}{c} -a_{3}\\ \begin{array}{c} 0\\ a_{1} \end{array} \end{array}\begin{array}{c} a_{2}\\ \begin{array}{c} -a_{1}\\ 0 \end{array} \end{array}\right)$$

$R^{*}$ is reduced to

(10) $$R^{*}=|\begin{array}{ccc} \cos\theta +a_{1}{}^{2}\left(1\;-\;\cos\theta \right) & a_{1}a_{2}\left(1\;-\;\cos\theta \right)\;-\;a_{3}\sin\theta & a_{1}a_{3}\left(1\;-\;\cos\theta \right)\;+a_{2}\sin\theta \\ a_{1}a_{2}\left(1\;-\;\cos\theta \right)\;+\;a_{3}\sin\theta & \cos\theta \;+\;a_{2}{}^{2}\left(1\;-\;\cos\theta \right) & a_{2}a_{3}\left(1\;-\;\cos\theta \right)\;-\;a_{1}\sin\theta \\ a_{1}a_{3}\left(1\;-\;\cos\theta \right)\;-\;a_{2}\sin\theta & a_{2}a_{3}\left(1\;-\;\cos\theta \right)\;+\;a_{1}\sin\theta & \cos\theta \;+\;a_{3}{}^{2}\left(1\;-\;\cos\theta \right) \end{array}|$$

where *E* is the third-order unit matrix, and the second term of the formula is a tensor product. The result is a matrix of three rows and three columns, and the rotation matrix *R* of 3 × 3 order is obtained by operation.

The ICP algorithm can theoretically find the optimal rotation matrix R* from the nearest point correspondence of the points; however, due to the low overlap between the source and target point clouds and the lack of noise immunity, it is easy to get the local optimal solution, leading to a mismatch. Therefore, we calculated the angle of the normal vector of the opposite face of the building to obtain the rotation matrix R* for rotation correction. The diagram of the result after rotation correction is shown in Figure 6c. After rotation correction, the normal vector of the opposite facade was parallel, but the translation needed to be further refined to get the result of Figure 6d. Therefore, for the next process, the aim was to determine the translation vector t*.

### 2.5. Final Shift Correction

**Figure 7:**
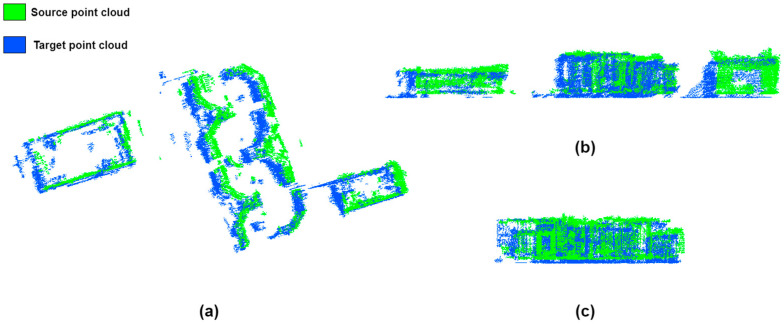
The source and target point clouds after coarse alignment and rotation correction are shown in green and blue: (**a**) point cloud of the top view; (**b**) point cloud of the side view; (**c**) point cloud of the front view.

The point clouds after PCA-based coarse alignment and rotation correction are shown in Figure 7a. The rotation errors of the two point clouds were accurately corrected, while the final displacement vector t* still needed to be accurately calculated. Figure 7b,c show the height difference between the two point clouds, and the z′ value of displacement vector t* could be calculated by calculating the height difference between the target and source point clouds of the building facade.

**Figure 8:**
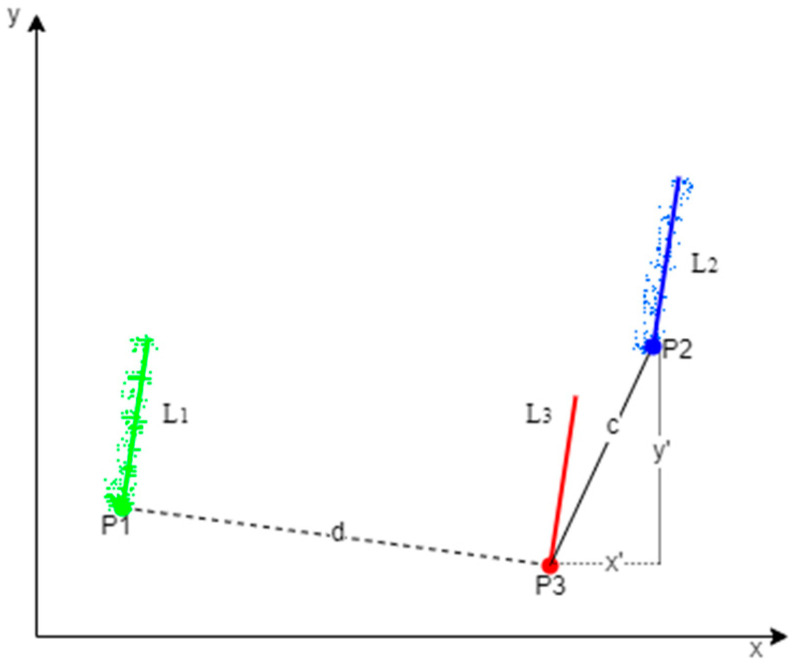
Schematic diagram of 2D displacement vector calculation for fine displacement of a pair of opposing facades of a building as an example.

For the two-dimensional displacement vectors x′ and y′, the point cloud of the building facade needed to be projected onto the two-dimensional plane for calculation. Using a priori information about the building to determine the vertical distance between the opposing facades of the building, we could determine d by measuring on Google Earth or in the field. As shown in Figure 8, we selected a set of building opposing facades of the experimental data to calculate the 2D displacement vectors, with the source and target point clouds corresponding to the two facades of the building. The point clouds of the building facades were projected to the xy plane, and the projected facade point clouds were fitted with RANSAC.

## 3. Results and Discussion

**Figure 9:**
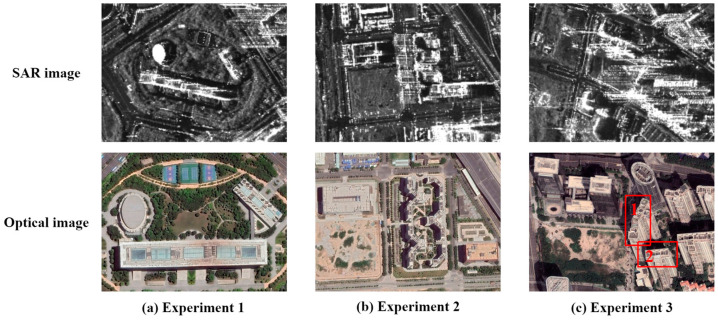
SAR images and optical images of three experimental scenes. The experimental area is red wireframe building 1 and building 2.

In this section, three sets of experimental data with different scenes are used to evaluate the performance of the proposed method in this paper. The TomoSAR point clouds in the experiments were generated by 3D imaging of the ascending and descending orbits of TerraSAR-X spotlight data in Baoan District, Shenzhen, with 18 images of the ascending orbit and 37 images of the descending orbit, both of which had a time span greater than 800 days. Three urban scenes with different complexity were selected for experiments, as shown in Figure 9. Experiment 1 and Experiment 2 verified the robustness of this paper’s method to align the ascending- and descending-orbit TomoSAR point clouds, while Experiment 3 verified the robustness of this paper’s method to align the single-orbit TomoSAR and MLS point clouds.

The alignment accuracy evaluation measured the angular rotation deviation and translation deviation between the aligned point cloud and the true point cloud. Due to the lack of real point clouds, we selected the TomoSAR point clouds generated when the geocoded distance direction and azimuth direction fitting error were less than 1 as the validation data for Experiments 1 and 2. The low sparse density of the TomoSAR point cloud and the small amount of information for extracting conjugate features did not allow the introduction of line, surface, and body-based feature elements for accuracy evaluation. Therefore, the alignment accuracy was evaluated by the difference between the calculated transformation parameters $E^{r}$ and $E^{t}$ and the validation data, according to the RMSE. In addition, for Experiment 3, which lacked validation data, we evaluated the difference between the angle θ of the two normal vectors between each facade shown in Figure 6, as well as the difference between the vertical distance of the outer endpoints of the aligned facade shown in Figure 8 and the true value d.

### 3.1. Homologous TomoSAR Point Cloud Alignment Experiment 1

In Experiment 1, an open urban area with no high-rise buildings around was selected; the main building in the area had 10 floors, and the building height was about 50 m. The descending-orbit TomoSAR and ascending-orbit TomoSAR point clouds were selected as the source and target point clouds, respectively. The two point clouds were extracted by statistical filtering and DoPP-based building points, and then, PCA-based coarse alignment was used; the coarse-aligned point clouds had good initial positions. Then, the point clouds were finely aligned using the method of this paper, ICP algorithm and FPFH algorithm, after which the results of the alignment were compared and evaluated in terms of accuracy.

**Figure 10:**
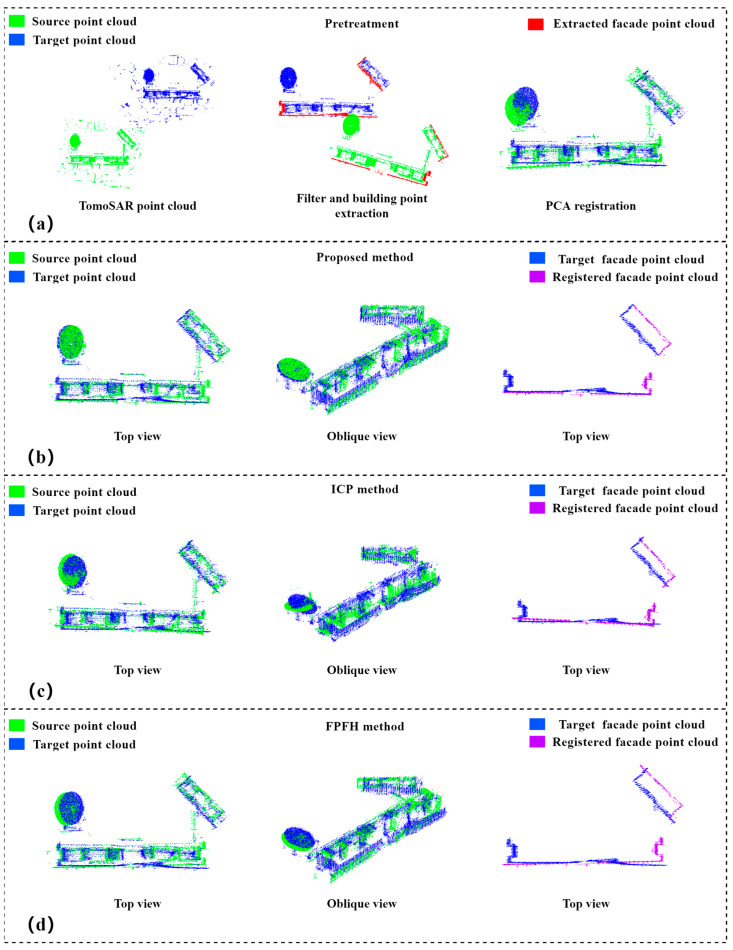
Alignment results of homologous TomoSAR point cloud of Experiment 1: (**a**) raw data and preprocessing of experimental data, including statistical filtering, extraction of DoPP-based facade points, density clustering, and PCA-based coarse alignment; (**b**) top and oblique views of the alignment results of the method in this paper and top view of the aligned facade points; (**c**) top and oblique views of the alignment results of ICP algorithm and top view of the aligned facade points; (**d**) top and oblique views of the alignment results of FPFH algorithm and top view of the aligned facade points.

In our experiments, the elevation information of the source and target point clouds were used for the fine alignment of the whole building point clouds; therefore, in the data preprocessing step, we extracted and filtered out the available elevation information and showed it with red point clouds in Figure 10a.The ICP algorithm was more sensitive to the initial position and rotation error of the point clouds, and the algorithm combined the two point clouds through the nearest neighbor search; The FPFH algorithm aligned two point clouds together by calculating the neighborhood features of the points. Since the TomoSAR point cloud is a sparse point cloud with an uneven distribution, the algorithm is limited by the process of feature extraction; however, due to the overlap of the two point clouds being extremely low, the ICP algorithm tended to obtain a local optimal solution, which led to unstable results of the alignment. Table 1 presents the quantitative evaluation results, revealing that the two point clouds still had large rotation and translation errors after coarse alignment. Although the ICP algorithm reduced the translation error, the rotation error increased due to its instability. The FPFH algorithm reduced the translation error and rotation error, but their values are still large. Our method could achieve a rotation error of 0.019° and a translation error of 0.1242 m, which achieved a good alignment.

For the alignment of the ascending and descending TomoSAR point clouds, we also calculated the root-mean-square error to evaluate the alignment results, and the RMSE was used to measure the deviation between the observed and real values. The distance between the aligned point cloud and the real value was greater than 6 m. Although the FPFH algorithm reduced the registration error, its RMSE is still 4.5473 m. As shown in Figure 10c,d, the aligned point cloud of the building facade still had a large deviation from the real building. The ICP algorithm was weakly applicable to the lift-track TomoSAR point cloud. The FPFH algorithm has some applicability to the homogenous TomoSAR point cloud, but it was limited by the quality of the point cloud itself, and its applicability was reduced for TomoSAR point cloud with fractures and uneven distribution. The main reason for its non-applicability was that the TomoSAR point cloud was not homogeneous, and the overlap rate of the two points was too low. The method in this paper does not use the overlap information for alignment but uses the elevation information for alignment; the results of the alignment are shown in Figure 10b. From the top and oblique views, the aligned point cloud and the target point cloud showed the structure of the building accurately, and the elevation point cloud also had a precise position. Since the TomoSAR point cloud contains many noise points and the TerraSAR-X image has a 3D resolution of 0.25 m, the alignment error of about 0.25 m is within the controllable range because the method in this paper has a high alignment accuracy and strong robustness for aligning the ascending and descending TomoSAR point clouds.

### 3.2. Homologous TomoSAR Point Cloud Alignment Experiment 2

A denser building complex was selected for Experiment 2. The main building in this area had 18 floors and was about 65 m high. The descending-orbit TomoSAR and ascending-orbit TomoSAR point clouds were selected as the source and target point clouds, respectively. Since the region was more complex compared with the scene of Experiment 1, the filtered point cloud still had a small number of dense outliers. Due to the limitation of the onboard TerraSAR-X incidence angle, the two TomoSAR elevation point clouds extracted using DoPP could not represent the facades of the six buildings in the scene, and the point cloud on the facade of the rightmost rectangular building in the scene has been removed because they were too sparse, but this did not affect the calculation of the method in this paper; accordingly, the extracted building facades were sufficient to complete the calculation of the rotation matrix and translation matrix.

**Figure 11:**
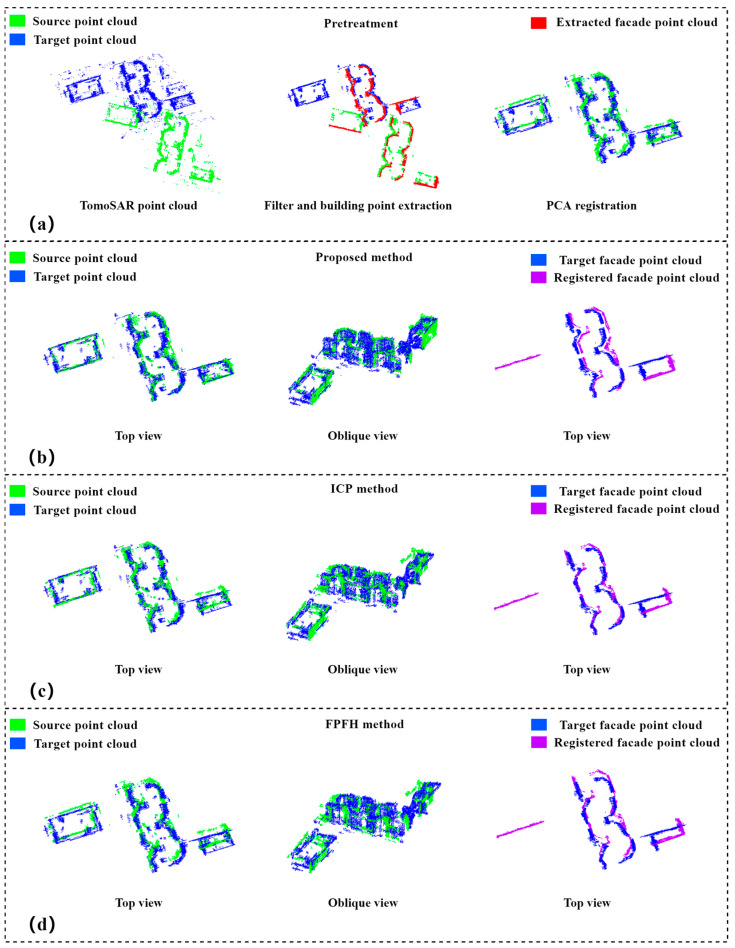
Alignment results of homologous TomoSAR point cloud in Experiment 2: (**a**) preprocessing of raw and experimental data, including statistical filtering, DoPP-based extraction of elevation points, density clustering, and PCA-based coarse alignment; (**b**) top and oblique views of the alignment results of the method in this paper and top view of the aligned facade points; (**c**) top and oblique views of the alignment results of ICP algorithm and top view of the aligned facade points; (**d**) top and oblique views of the alignment results of FPFH algorithm and top view of the aligned facade points.

The coarse alignment based on PCA roughly aligned the two point clouds together according to their principal axes, and the two point clouds after the coarse alignment also had certain rotation and translation errors. As in Experiment 1, we used the method of this paper, ICP and FPFH, to finely align them. From the top and oblique views in Figure 11b–d, our method achieved more accurate alignment results than the ICP and FPFH algorithm, and the purple building elevation points were parallel to the source building facade points and had precise spatial positions after alignment. As shown in Table 2, the alignment results of the ICP and FPFH algorithm also had large rotation and translation errors, while the errors of the methods in this paper were less than 0.25 m, demonstrating the high accuracy and robustness of the TomoSAR point cloud alignment.

The results of Experiments 1 and 2 demonstrate the high accuracy and strong adaptability of the method in this paper for aligning homologous TomoSAR point clouds, as well as the good robustness of the alignment using the characteristics of the building facade for the very low overlap rate of the two point clouds. The limitations of the image data and building environment led to the point cloud of a particular track not being able to support it for alignment. Our experimental solution was to align the TomoSAR point cloud on one side with the point cloud on one side scanned by other sensors.

### 3.3. Cross-Source TomoSAR Point Cloud Alignment Experiment

Experiment 3 selected the TomoSAR point cloud obtained from 38-view downlinked TerraSAR-X data after 3D imaging and the point cloud of one side of the building scanned by a ZEB-REVO RTT portable laser scanner of CHC NAVIGATION. The experimental area was a high-rise building with a complex building environment, and the main building had 32 floors and was about 100 m high. The downlinked TomoSAR and MLS point clouds were selected as the source and target point clouds, respectively. Since the density of the MLS point cloud was denser than that of TomoSAR point cloud, the threshold value for setting filtering and building facade point extraction needed to be increased.

**Figure 12:**
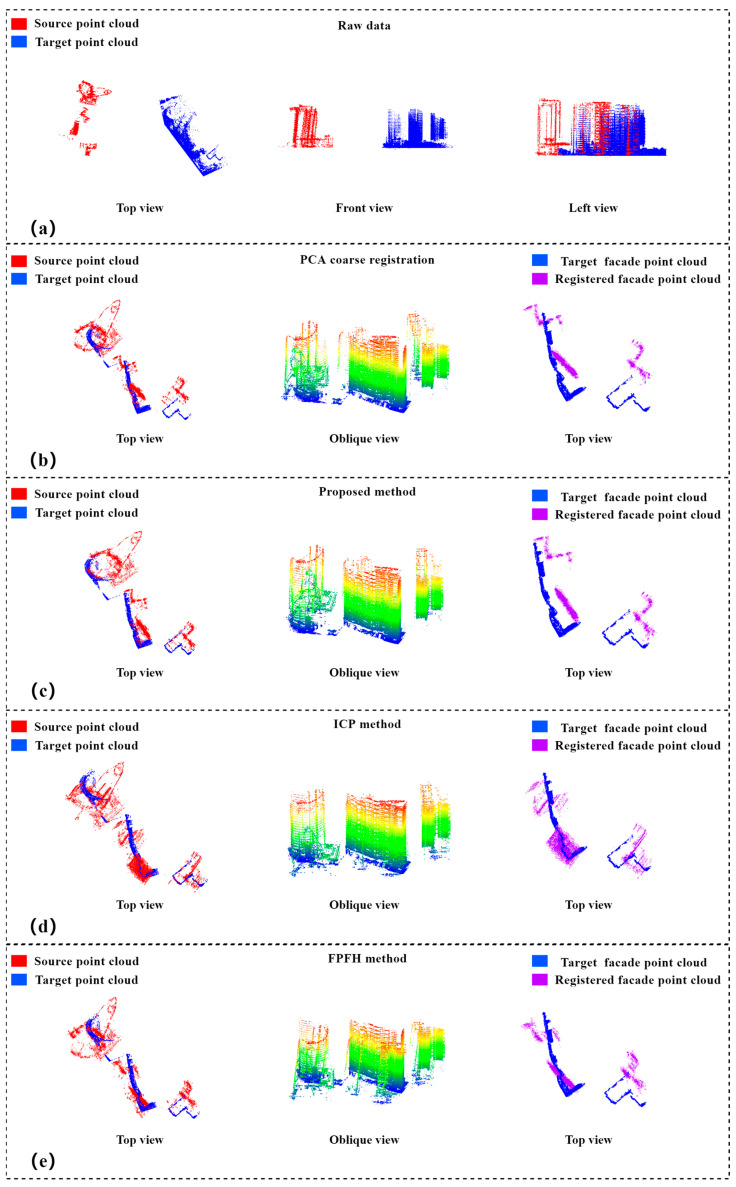
Experiment 3 alignment results of cross-source TomoSAR point cloud with MLS point cloud: (**a**) top, front, and left views of the original data; (**b**) top and oblique views of the coarse alignment results based on PCA and top view of the aligned facade points; (**c**) top and oblique views of the alignment results of this paper and top view of the aligned facade points; (**d**) top and oblique views of the alignment result of ICP algorithm and top view of the facade point after alignment; (**e**) top and oblique views of the alignment results of FPFH algorithm and top view of the aligned facade points.

The results based on PCA coarse alignment are shown in Figure 12b. From the top and oblique views, the two point clouds after aggregation had obvious rotation and translation errors, and the top view of the building facade points showed the spatial position relationship between the aligned facade point cloud and the target point cloud facade point cloud, whereas the coarsely aligned facade points did not show their spatial position correctly and could not express the building structure in the top view. In Experiments 1 and 2, the method of this paper and the ICP algorithm were used to finely align the point clouds. The alignment results of the ICP and FPFH algorithm are shown in Figure 12d,e, where the two point clouds showed visually obvious rotation errors, and the two facade points did not have correct spatial positions. The alignment results of the method in this paper are shown in Figure 12c. From the top and oblique views of the two points, the two point clouds correctly represented visually the buildings for which the accuracy was evaluated, and the facade points also had the correct spatial positions.

In the absence of real validation data, we evaluated the alignment results by the angular difference θ of the designed facade normal vectors and the outer endpoint Δd of the facade. We calculated θ and Δd for planar buildings 1 and 2 within the scene in Figure 9c after alignment by the ICP algorithm, FPFH algorithm and the method in this paper, and the results are shown in Table 3. The ICP method could not correctly rotate the two point clouds, and the angle between the opposite elevations of buildings 1 and 2 after alignment was about 24°, while the value of Δd was greater than 1.5 m. The FPFH method also could not rotate the two point clouds correctly, after registration, the relative elevation angle of building 1 and 2 is about-19°, and the Δd value is more than 3.0 m. From the experimental results and accuracy analysis, the ICP and FPFH algorithm could not calculate the exact correspondence between the TomoSAR point cloud and the MLS point cloud, and although the translation error was close to the meter level, it had a more obvious rotation error. However, our proposed method had significantly improved accuracy compared with the ICP and FPFH algorithm; the translation error reached 0.25 m, and the normal vectors of the rotated building facade point clouds were parallel with minimal rotation error. Since the TomoSAR and MLS point clouds contained many noise points and the difference between the two point clouds was too large, we believe that the error after alignment is within the acceptable range, but the accuracy and efficiency of the alignment still have room for improvement; in particular, the efficiency of the algorithm needs to be further optimized.

### 3.4. Discussion

In this paper, we proposed an alignment method to align homologous and cross-source TomoSAR point clouds using the normal vectors and outer endpoints of building facades. The above experimental results verified the effectiveness of the proposed method. Compared with the famous PCA, ICP, and FPFH algorithms, the proposed method has the following advantages and disadvantages:

(1) The most important significance of our method is that it could be applied to both homologous and cross-source TomoSAR point cloud registration, helps to accurately correct rotation and translation errors, and could realize the complete observation of 3D buildings based on TomoSAR point clouds. In contrast, PCA algorithm could only achieve rough registration of two point clouds, and subsequent fine alignment was required to obtain more accurate alignment results. Although ICP algorithm can reduce the translation error, the registration result had a large rotation error and could not correctly display the building structure. The FPFH algorithm was applicable to homologous TomoSAR point clouds in simple environments, but it was not used in homologous TomoSAR point clouds with complex environments and cross-source TomoSAR point clouds.

(2) Our method does not depend on the overlap between the two point clouds but obtains the architectural points in the experimental scene and extracts the facade points through statistical filtering and DoPP projection filtering and calculates the rotation matrix by using the angle of the normal vector of the opposite side of the building and then uses the outer endpoint of the building facade projection to estimate the fine translation. The experimental results and actual data show that the method proposed in this paper had higher accuracy than other algorithms.

(3) However, the engineering process of this paper is more complex complicated and less time-efficient, especially in the extraction of building facade information. Furthermore, the calculation of facade information consumes most of the time. In addition, we need to measure the vertical distance between opposite building elevations from high-precision remote sensing images, cadastral information, or in the field.

In summary, for satellite-based synthetic SAR tomography point clouds, the method in this paper can achieve the alignment of their homologous or cross-source urban multi-view point clouds using building facade information. Moreover, we will continue to refine the method and apply it to the alignment of point clouds acquired by other sensors of different quality.

## 4. Conclusions

The TomoSAR point cloud of a single track cannot show the complete building structure. In order to solve this problem, this paper proposed a robust homologous and cross-source TomoSAR point cloud registration method. Under the condition of many noise points and a low overlap rate, a complete TomoSAR point cloud registration process was designed and implemented. The experimental process includes statistical filtering, building facade point extraction based on DoPP, density clustering, and rough registration based on PCA. The final rotation and translation coefficients are calculated from the angle of the normal vector of the building facade and the distance between the outer endpoints. Experimental results showed that, compared with the ICP algorithm, the proposed method is more robust in registering homologous and cross-source TomoSAR point clouds.

However, there are several aspects of our work that can be improved. First of all, for the facade of building facade points, the method of this paper depends on the selection of parameters, which greatly reduces the efficiency of registration. When there are enough spaceborne TomoSAR point cloud data, we can use some deep learning methods to classify and segment them. Secondly, the method in this paper can be applied to urban point cloud registration collected by sensors of different quality. In future work, we will optimize the efficiency of this method and further analyze and evaluate its performance of this method.

## Figures and Tables

**Table 1:** Alignment accuracy parameters of Experiment 1.

Method	$\mathrm{Rotation}\;\mathrm{Error}\;E^{r}$ (°)	$\mathrm{Translation}\;\mathrm{Error}\;E^{t}$ (m)	RMSE (m)
PCA	15.0321	9.2350	8.9866
ICP	16.1078	6.7562	6.9740
FPFH	9.2041	2.4132	4.5473
Proposed method	0.0189	0.1242	0.1913

**Table 2:** Alignment accuracy parameters of Experiment 2.

Method	$\mathrm{Rotation}\;\mathrm{Error}\;E^{r}$ (°)	$\mathrm{Translation}\;\mathrm{Error}\;E^{t}$ (m)	RMSE (m)
PCA	12.0547	8.9407	8.6295
ICP	8.1752	6.8431	7.0945
FPFH	6.0982	9.8456	8.7361
Proposed method	0.0107	0.1584	0.1802

**Table 3:** Alignment accuracy parameters of Experiment 3. Processing times are obtained by a regular desktop PC (Intel i7-11700).

Building	Method	Θ (°)	Δd (m)	Time (s)
Building one	ICP	24.5624	1.5898	4.1847
	FPFH	−19.4571	3.2489	64.4830
	Proposed	0.1681	0.2259	24.4830
Building two	ICP	24.2873	1.5788	3.7857
	FPFH	−19.6317	3.3115	47.6954
	Proposed	0.1487	0.2314	22.8704

## Data Availability

Not applicable.
